# Special Issue: Biopolymer-Based Materials for Biomedical Engineering

**DOI:** 10.3390/ma15082942

**Published:** 2022-04-18

**Authors:** Joaquim M. Oliveira, Viviana P. Ribeiro, Rui L. Reis

**Affiliations:** 1 3B’s Research Group, I3Bs—Research Institute on Biomaterials, Biodegradables and Biomimetics of University of Minho, Headquarters of the European Institute of Excellence on Tissue Engineering and Regenerative Medicine, AvePark, Parque de Ciência e Tecnologia, Zona Industrial da Gandra, Barco, 4805-017 Guimarães, Portugal; 2 ICVS/3B’s—PT Government Associate Laboratory, 4710-057 Braga, Portugal

In the field of tissue engineering and regenerative medicine (TERM), the use of traditional biomaterials capable of integrating the host tissue to promote the healing and regenerative process while it degrades has become less and less a focus of inspiration. The current trend is to increase the complexity of the host materials in order to better emulate the extracellular microenvironment of heathy and disease tissues [1]. Thus, the combination of materials engineering with other emerging fields, such as nanotechnology, cell and molecular therapy, and precision medicine, can allow for the development of innovative biopolymer-based scaffolds for specific biomedical approaches [2,3,4,5]. New and recent insights in bioprinting, reverse engineering, and image acquisition are an asset for advanced scaffolds design and biofabrication. Moreover, it has been recognized that the emulation of biological and mechanical diversity of in vivo tissues can be best achieved by the exploitation of natural and/or synthetic biopolymers combined with these emerging technologies [6,7].

This Special Issue covers the abovementioned subjects with the main goal of collecting significant contributions related with biopolymer-based materials applied in biomedicine and TERM, as well as the emerging scaffolding strategies and manufacturing techniques used for biomaterials processing. Nano-tools for biopolymers functionalization and materials-cells interactions were also explored. The current concerns related to the use of sustainable polymer sources and processing techniques allowed us to increase the research studies in this field (especially when designed for biomedical applications). Thus, we can state that our efforts were quite successful, and the proposed multidisciplinary topic resulted in six published papers briefly summarized below.

An injectable thermosensitive hydrogel was produced by Youn et al. [8] as a drug and cell delivery system. The composite hydrogel matrix was prepared by mixing pluronic^TM^ F-127 (PF) and silk fibroin (SF) in an aqueous solution and testing the mixture at different ratios. The PF provided a platform for the entire hydrogels’ support, whereas SF enhanced the structure by the intermolecular interactions promoted by the physical cross-linking. Authors showed that at proper amounts, SF improved the mechanical strength and decreased the degradation rate of the hydrogels improving the drug release rate of hydrophobic drugs. Moreover, the presence of SF also reduced the cytotoxicity of the hydrogels induced by the PF. Thus, authors confirmed that the injectable PF-SF hydrogels are promising for future tissue regeneration applications.

The synergistic effect of human interferon α2 (IFNα2) and thymosin α1 (Tα1) proteins, commonly used for the treatment of viral infections and cancer, were innovatively explored by Aslam et al. [9] by genetic fusion of IFNα2–Tα1 genes in a single molecule. The recombinant IFNα2–Tα1 exhibited elevated anticancer and antiviral activities as compared to IFNα2 alone used as control. These results were confirmed by in vitro analysis, in which the IFNα2–Tα1 was genotoxic and more efficient in inhibiting cell growth as compared to IFNα2 alone. Molecular analysis revealed that IFNα2–Tα1-treated cells expressed higher levels of proapoptotic genes and HCV replication inhibitor genes as compared to the IFNα2-treated cells. Despite the fact that in vivo trials are needed to further explore the pathways responsible for the combined antiviral and anti-cancer activity of IFNα2–Tα1, the present findings confirmed the synergistic effect of IFNα2 and Tα1 and their potential as combined therapies.

Su et al. [10] tested the wear rate of different materials typically used for the femoral head and acetabular liner in hip joint prothesis. A hip joint simulator was used for testing the wear rate between different friction pairs in order to determine the suitable prosthesis according to different processing technologies and costs. Different materials had distinct wear rate efficacies. However, the combination of cobalt-chromium-molybdenum alloy (CoCrMo) femoral head with highly cross-linked polyethylene (XLPE) liner showed superior wear resistance and cost-effectiveness as hip prosthesis as compared to other contact materials.

RGD peptide-conjugated chitosan (CT) hydrogels were proposed by Chen et al. [11] as an alternative to the endobarrier medical device used in the small intestines for the treatment of type 2 diabetes and obesity. The RGD-CT hydrogels were demonstrated to be highly biocompatible and non-cytotoxic in vitro and were effectively retained in the small intestine of rats, inducing a significant decrease of body weight while the blood and hematic biometrics were maintained at normal levels. The authors also consider the RGD-CT gels as patient-specific anti-obesity therapies, due to the possibility of adjusting the oral intake of the RGD’s according to patients’ needs.

Novel cell-penetrating peptides (CPPs) were investigated by Liu et al. [12] in order to enhance the endosome escaping ability of CPPs promoted by histidine. Previously, authors showed that a CPP peptide called RALA (arginine-rich) presented suitable transfection efficacy and potential clinical use. However, RALA peptide contains only one histidine in each chain, which led the authors to develop these new peptides named HALA with an increased histidine ratio. Transfection results revealed superior outcomes for the HALA peptides. Moreover, multiple pathways led to a mechanism of endocytosis being revealed by pDNA nanocomplexes, wherein caveolae played the main role. All combined, a novel peptide-HALA2 was discovered with high cellular transfection efficacy which ultimately can be applied for gene therapy.

Finally, Hao et al. [13] presented a new type of electroprobe made in biocompatible natural materials for neuronal tissue engineering applications. The neuroelectrode-associated conductive biomaterials presented good biocompatibility and a gradient microstructure for cell viability, growth, and neurons bonding in vivo. Moreover, the developed bio-electro probe presented an elastic modulus closer to that observed for natural brain barrier and a superior conductivity as compared to metal electrodes. The study represents the first research line for long-term studies of neural electrodes implantation in cortical nerve with more efficient signaling transmission.
